# Should Skin Biopsies Be Performed in Patients Suspected of Having Parkinson's Disease?

**DOI:** 10.1155/2017/6064974

**Published:** 2017-10-30

**Authors:** Timo Siepmann, Ana Isabel Penzlin, Ben Min-Woo Illigens, Heinz Reichmann

**Affiliations:** ^1^Department of Neurology, University Hospital Carl Gustav Carus, Technische Universitaet Dresden, Dresden, Germany; ^2^Department of Neurology and Rehabilitation, Klinik Bavaria Kreischa, Kreischa, Germany; ^3^Department of Neurology, Beth Israel Deaconess Medical Center, Harvard Medical School, Boston, MA, USA

## Abstract

In patients with Parkinson's disease (PD), the molecularly misfolded form of *α*-synuclein was recently identified in cutaneous autonomic nerve fibers which displayed increased accumulation even in early disease stages. However, the underlying mechanisms of synucleinopathic nerve damage and its implication for brain pathology in later life remain to be elucidated. To date, specific diagnostic tools to evaluate small fiber pathology and to discriminate neurodegenerative proteinopathies are rare. Recently, research has indicated that deposition of *α*-synuclein in cutaneous nerve fibers quantified via immunohistochemistry in superficial skin biopsies might be a valid marker of PD which could facilitate early diagnosis and monitoring of disease progression. However, lack of standardization of techniques to quantify neural *α*-synuclein deposition limits their utility in clinical practice. Additional challenges include the identification of potential distinct morphological patterns of intraneural *α*-synuclein deposition among synucleinopathies to facilitate diagnostic discrimination and determining the degree to which structural damage relates to dysfunction of nerve fibers targeted by *α*-synuclein. Answering these questions might improve our understanding of the pathophysiological role of small fiber neuropathy in Parkinson's disease, help identify new treatment targets, and facilitate assessment of response to neuroprotective treatment.

## 1. Introduction

Clinical management of Parkinson's disease (PD) has undergone substantial innovations that comprise both diagnostic assessment and pharmacotherapy. However, the disease still poses a clinical challenge due its high prevalence and our restricted understanding of its underlying pathology. The latter explains why, to date, no causative treatment is available. Moreover, the range of tools to detect early and premotor pathology is limited. This may prevent timely initiation of dopaminergic treatment and thereby improvement of quality of life [1]. In order to provide individualized treatment, it is also important to differentiate PD from atypical Parkinsonism syndromes, a problem which is difficult to solve in clinical practice, particularly in early and prodromal disease stages. In an effort to address these challenges research has focused on identifying reliable disease markers. The molecularly misfolded form of the protein *α*-synuclein has recently been identified in autonomic nerve fibers of the skin. In these small lightly myelinated and unmyelinated nerve fibers, *α*-synuclein deposits are present even in the early stages of PD as demonstrated by an analysis of skin biopsies immunohistochemically costained for *α*-synuclein and nerve fibers [2]. This technique has enabled, for the first time, assessment of synucleinopathic changes in the peripheral nervous system and has been reproduced or modified in several research studies in patients with PD [3–5]. However, the mechanisms whereby synucleinopathic nerve damage relates to central neurodegeneration in later life and how it might play a causative role in PD remain unknown.

Immunohistochemical detection of intraneural *α*-synuclein deposition in epidermal structures innervated by autonomic small fibers offers a novel tool to study pathology in PD and might provide a valid biomarker of PD as suggested by a number of well-designed experimental studies [2, 6, 7]. We reviewed the current literature on detection of *α*-synuclein in skin biopsies in order to approach the question whether this technique might be a useful supplement to other clinical diagnostic tests in patients with possible Parkinson's disease.

## 2. Synucleinopathic Small Fiber Neuropathy: What Do We Know?

The epidermal layer of the skin is innervated by small fibers, which comprise unmyelinated C-fibers and thinly myelinated A-delta fibers [8]. A structural analysis of cutaneous denervation in a population of PD patients has displayed a link between *α*-synuclein deposition in autonomic small fibers and severity of clinical symptoms related to autonomic dysregulation such as orthostatic hypotension or sweating disturbances, indicating the usefulness of skin pathology as potentially valid disease marker [2]. Additional analyses within the same cohort have shown that, in patients with PD, the amount of *α*-synuclein positive nerve fibers normalized to total intraepidermal nerve fiber density (*α*-synuclein ratio) is enhanced in both sympathetic cholinergic (sweat gland innervating) and sympathetic adrenergic (pilomotor muscle innervating) nerve fibers. Interestingly, higher *α*-synuclein ratios were also associated with higher severity of motor symptoms in this study. However, if and to what degree this positive correlation might point to a direct causative association between peripheral autonomic and central motor pathology remains unknown. An indirect indication toward such an association has been provided by the similar morphology of misfolded *α*-synuclein accumulation in cutaneous small fibers and in neurites in the substantia nigra. In both structures, *α*-synuclein staining showed an irregular line following the course of the neural structure (small fiber and neurite, resp.) [6].

Subsequent investigations have corroborated these observations and provided Class III evidence. An analysis of skin biopsies detected *α*-synuclein aggregation in skin samples from all 21 patients with PD but no aggregation in any of the samples from the 20 individuals with Parkinsonism syndromes (i.e., vascular Parkinsonism, tauopathies, and pathogenic Parkin mutations) [9]. The study was limited by the lack of quantitative analysis of intraneural *α*-synuclein load and the absence of normalization to intraepidermal nerve fiber loss. Its strength, however, was the use of an antibody targeting the presumably pathogenic misfolded (phosphorylated) form of *α*-synuclein. Another study used skin biopsies with quantification of *α*-synuclein deposition in pilomotor and sudomotor nerve fibers normalized to the overall intraepidermal nerve fiber density. This analysis demonstrated >90% sensitivity and >90% specificity to distinguish PD patients from control individuals via *α*-synuclein ratios in a prospective longitudinal study [7].

## 3. Cutaneous *α*-Synuclein in Prodromal Disease Stages/Possible PD

Since phosphorylated *α*-synuclein is present in patients in PD even in the early stages of the disease (Hoehn and Yahr I and II), research has recently addressed the question whether this pathology might also be present in the premotor stages. Skin nerve pathology was assessed in individuals with REM sleep behaviour disorder (RBD) as these patients display a substantially increased risk of developing PD in later life. The likelihood of developing PD was further determined based on additional predictors such as reduced dopamine transporter binding in FP-CIT-SPECT and anosmia. Consistent associations between *α*-synuclein deposition and presence of RBD, dopamine transporter insufficiency, and olfactory dysfunction were observed [10]. In line with this observation, a recent study in 12 patients with polysomnographically confirmed RBD and 55 sex- and age-matched healthy controls has provided Class III evidence that intraneural deposition of phosphorylated *α*-synuclein is present in patients with RBD [11].

These findings indicate that cutaneous phosphorylated *α*-synuclein might help to identify individuals with prodromal Parkinson's disease which might be particularly important in the development of novel disease modifying strategies. Although promising, these results need to be considered with care since only 18 patients with RBD were included in the analyses. Longitudinal data is warranted to assess whether phosphorylated *α*-synuclein might indeed predict conversion from RBD into PD. Additionally, experiments need to be repeated in a larger study population to allow evaluation of external validity.

## 4. Distinguishing Synucleinopathies and Nonsynucleinopathic Neurodegenerative Disorders

Among the potential clinical applications of cutaneous *α*-synuclein assessment, its capacity to differentiate patients with PD from patients with other synucleinopathies based on distinct patterns of neural skin pathology has been discussed. In a cross-sectional investigation, 62% of skin biopsies from PD patients showed cutaneous *α*-synuclein, whereas only 7% of skin biopsies from patients with atypical Parkinson syndrome were positive for *α*-synuclein. However, this study considered *α*-synuclein inclusions in the epidermis and in pilosebaceous units but did not specifically assess intraneural accumulation [12]. Further limitations include a relative small sample size (34 subjects with PD, 33 patients with atypical Parkinson syndrome, and 20 control individuals) and heterogeneity within the group of atypical Parkinson syndrome which included synucleinopathies, such as dementia with Lewy bodies (DLB), multisystem atrophy (MSA), and tauopathies such as Alzheimer's disease (AD) and progressive supranuclear palsy (PSP), possibly jeopardizing interpretability.

Synucleinopathic neurodegenerative disorders have been hypothesized to display distinct patterns of *α*-synuclein aggregates in the central and the peripheral nervous system. Contrarily to PD, MSA has been described as isolated disorder of the central nerve system with accumulation of phosphorylated *α*-synuclein limited to brain tissue and preganglionic neurons [13]. In line with this assumption, the examination of cutaneous autonomic adrenergic nerve fibers revealed no deposition of phosphorylated *α*-synuclein in MSA patients [14]. However, an immunohistochemical analysis of cutaneous somatosensory fibers has unexpectedly revealed enhanced *α*-synuclein deposition in sensory fibers in 67% of MSA patients, whereas a similar fraction of PD patients showed *α*-synuclein aggregation in autonomic small fibers [15]. In line with this observation, sudomotor denervation has been reported in patients with MSA indicating postganglionic impairment of the sympathetic nervous system which may contribute alongside degeneration of central autonomic structures to dysautonomia in these patients [16]. These observations modify the current conception of MSA as a solely or predominant disease of the central nervous system.

Further insights into the pathophysiology of synucleinopathies have been provided by Donadio et al. who found length-dependent somatic and autonomic small fiber damage, both in patients with PD and in those with pure autonomic failure (PAF). The degree of cutaneous denervation was associated with the amount of *α*-synuclein accumulation [17]. Interestingly, differences in patterns of cutaneous denervation and *α*-synuclein accumulation appear not to be related to patterns of autonomic dysfunction including cardiovascular dysfunction detected by head-up tilt-test and sympathetic nerve dysfunction characterized by microneurography [18]. These discrepancies between structural and functional changes of the autonomic nervous system among synucleinopathies underscore our still limited understanding of the underlying pathophysiological mechanisms [19]. However, it also implies that defining disease specific patterns of *α*-synuclein pathology might improve early diagnosis, help monitoring synucleinopathies in clinical practice, and facilitate assessment of response to disease modifying treatment. In order to improve our understanding of the direct effects of *α*-synuclein on skin nerves, to what extent structural changes of these nerves are related to their functional impairment of these fibers ought to be investigated. This highlights the need for detailed characterization of skin denervation patterns, including quantitative examinations of intraneural *α*-synuclein deposition beyond autonomic fibers in following investigations. Further research is needed to provide a detailed characterization of *α*-synuclein accumulation patterns among somatic and autonomic small nerve fibers in large populations of affected patients with synucleinopathies such as PD and MSA. This should also include longitudinal studies to characterize progression of neuropathy.

## 5. How to Obtain and Handle Skin Biopsies

**Figure 1:**
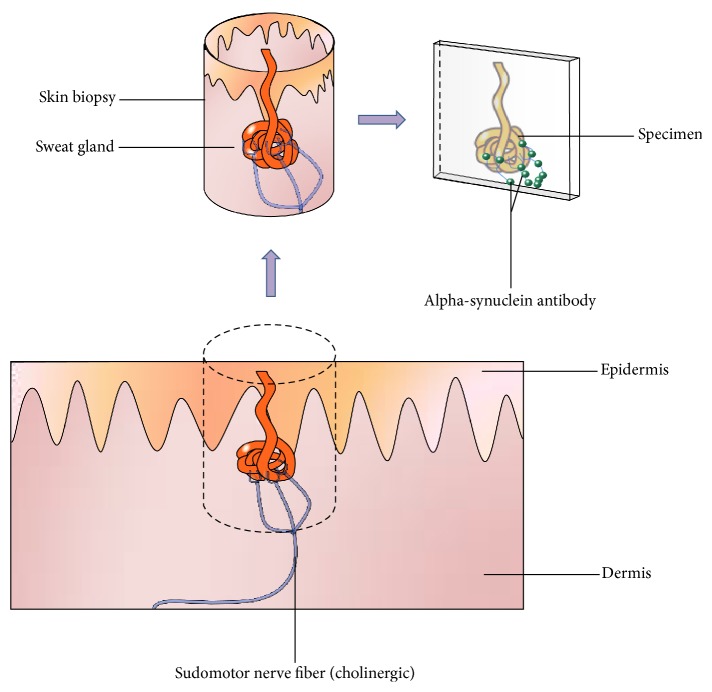
Obtaining a skin biopsy containing a sweat gland and staining of sudomotor nerve fibers. A punch biopsy is obtained and stained using immunohistochemical antibodies for *α*-synuclein (e.g., anti-phosphorylated *α*-synuclein antibody) and nerve fibers (anti-protein gene product 9.5 antibody). Additional costaining with the cholinergic marker vasoactive intestinal peptide can be performed to specifically identify *α*-synuclein deposits in cholinergic sudomotor fibers. Figure designed by Dr. Siepmann and Dr. Illigens.

The structural assessment of autonomic skin innervation is still on an experimental level. Both techniques to obtain and handle biopsies and procedures to process and analyze specimens require standardization. Proposed protocols include immunohistochemical analysis of phosphorylated *α*-synuclein or the *α*-synuclein/PGP-ratio in skin punch biopsies obtained from varying skin areas such as the dorsal forearms or the lower legs. Commonly, three-millimeter biopsies are obtained following local anesthesia with lidocaine (see Figure 1).

Biopsy specimens are then fixed (e.g., in Zamboni solution) and cryoprotected. Frozen tissue blocks are cut into sections using a microtome. Parameters and techniques vary among published protocols. Multiple costains with *α*-synuclein, phosphorylated *α*-synuclein, protein gene product (PGP) 9.5 (nonspecific nerve fiber marker), tyrosine hydroxylase (TH, adrenergic fiber marker), and vasoactive peptide (VIP, cholinergic fiber marker) allow analyses of *α*-synuclein deposits in different types of small fibers. To determine intraepidermal nerve fiber density PGP 9.5-positive fibers in skin biopsies should be counted in blinded fashion with results expressed as number of fibers crossing the dermal-epidermal junction per millimeter. Sweat glands and pilomotor muscles can be imaged and nerve fiber densities quantified, preferably in a blinded fashion. In sweat glands, the number of nerve fibers intersecting the circles within the area of interest is counted and reported as the percentage of circles with nerve fiber crossing. Similarly, in pilomotor muscles, the average number of nerve fibers intersecting horizontal lines across the width of the pilomotor muscle in 3 *μ*m thick confocal images is reported in fibers/mm. Techniques to assess *α*-synuclein deposition in vasomotor fibers are still being designed. Alpha-synuclein/nerve fiber density ratios can then be calculated for (a) epidermis, (b) sweat glands, and (c) pilomotor muscles [2, 7, 17]. Other approaches include advanced morphological qualitative analyses of *α*-synuclein deposition [6, 10].

## 6. Functional Assessment of Cutaneous Autonomic Denervation

Dysfunction of autonomic small fibers is of clinical relevance. In general, autonomic neuropathies constitute a group of conditions in which the small, unmyelinated, and lightly myelinated autonomic nerve fibers display structural and functional impairment. Autonomic symptoms involve the cardiovascular, urogenital, gastrointestinal, sudomotor, and pupillomotor systems. Clinical manifestations range from bladder dysfunction over orthostatic hypotension to dyshidrosis, erectile dysfunction, and obstipation. Although diabetes is the most prevalent cause of autonomic fiber dysfunction in more developed countries, targeted fiber damage can also occur in various systemic diseases such as amyloidosis, paraneoplastic syndromes, and infectious diseases and has been shown to be targeted by peripheral synucleinopathies such as PD [3, 18–20].

While structural assessment of *α*-synuclein pathology in skin biopsies is the most direct test of cutaneous *α*-synuclein pathology, evaluation of small fiber dysfunction resulting from intraneural *α*-synuclein deposition might be of additional diagnostic value. This rationale is indirectly supported by a strong correlation between *α*-synuclein load in cutaneous small fibers and measures of cardiovascular sympathetic and parasympathetic function, although these tests are composite measures of central and peripheral autonomic function and do not specifically capture small fiber integrity [2]. On these composite assessments, cutaneous *α*-synuclein deposits can be present even in PD patients without autonomic dysfunction. This raises the question if more specific tests of functional integrity of small autonomic fibers might be necessary in the assessment of cutaneous autonomic denervation compared to current cardiovascular tests. In fact, functional evaluation of autonomic cutaneous small fibers is of growing importance in the assessment of autonomic neuropathy [20]. A recent study has shown that pilomotor nerve fibers display functional impairment in early stages of PD which correlated with severity of autonomic symptoms [21]. However, skin biopsies have not been obtained in this study. Therefore, it remains speculative whether pilomotor dysfunction relates to *α*-synuclein deposition in these nerve fibers. It is however noteworthy that, among types of autonomic small fibers, pilomotor nerves displayed the highest concentration of intraneural *α*-synuclein, supporting the potential usefulness of pilomotor function assessment in the detection of early disease related changes [2].

**Figure 2:**
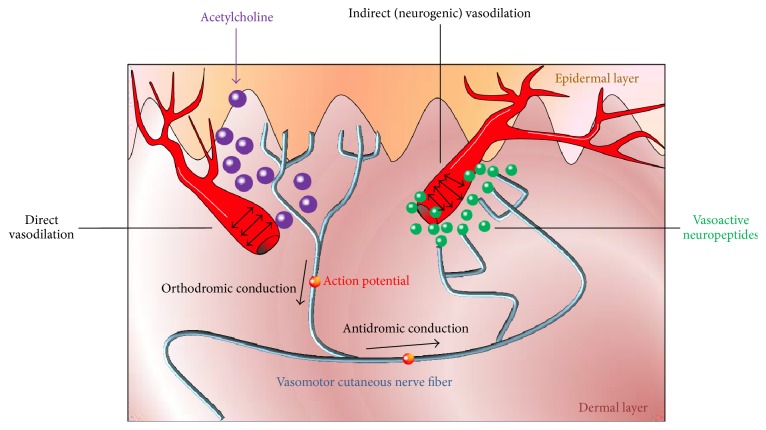
Illustration of the vasomotor axon reflex. Iontophoresis of acetylcholine induces vasodilation in the “direct” skin region of application via receptor activation. Consequently, an action potential emerges in the afferent nerve innervating this vessel. This potential travels in an orthodromic fashion to an axonal branch point where it switches to another vasomotor fiber. Upon antidromic conduction, the action potential reaches terminal nerve endings adjacent to a neighboring population of blood vessels. From these terminals, vasoactive substances are released to cause “indirect” vasodilation in a skin region which is surrounding the region of iontophoresis. Consecutive enhancement of blood flow relates to functional integrity of the stimulated vasomotor nerve fiber. Similarly, the axon reflex can be evoked in sympathetic adrenergic pilomotor and sympathetic cholinergic sudomotor fibers. Figure designed by Dr. Siepmann and Dr. Illigens.

Assessment of autonomic cutaneous small fiber function is based on quantification of axon reflex responses. Autonomic skin nerve fibers respond to mechanical, physical, and chemical stimuli with an axon reflex-mediated response. Local stimulation of the cutaneous vasomotor (blood vessel innervating) fibers by iontophoretic application of acetylcholine evokes orthodromic conduction of an action potential which then reaches an axon branch point. From this branch point, the potential is conducted antidromically to adjacent cutaneous blood vessels. These vessels are located in an “indirect” area surrounding the skin area where the axon reflex inducing stimulus is applied. There, activated vasomotor small fibers release vasoactive substances such as substance P and calcitonin gene related peptide to cause vasodilation and plasma extravasation [22] (Figure 2). This response can be quantified by laser Doppler assessment of blood flow increase in the indirect skin region [23, 24]. Similarly, functional integrity of sudomotor nerve fibers can be examined via imaging-based quantification of axon reflex responsivity to cholinergic stimulation (adrenergic stimulation in pilomotor fibers, resp.) [22, 25]. In addition to pilomotor dysfunction, sudomotor axon reflex responsiveness was shown to be reduced in PD [26]. Nevertheless, the degree of association between functional impairment and structural deterioration of the cutaneous small fibers has not yet been elucidated. Forthcoming such considerations, the reproducibility and external validity of quantitative tests of autonomic sudomotor and pilomotor functions in the evaluation of PD patients and its association with the *α*-synuclein load are currently being investigated in an ongoing longitudinal controlled multicenter trial [19].

## 7. Limitations and Challenges

While growing evidence emphasizes the diagnostic value of the assessment of *α*-synuclein pathology in PD, most of the available data is restricted to the detection of *α*-synuclein positive skin nerve fibers. Only few studies provide quantitative data on the extent of *α*-synuclein accumulation in sudomotor and pilomotor nerve fibers [2, 4]. Additionally, most of the available date is originated from single center studies and lack independent blinded evaluation.

Alpha-synuclein load in cutaneous small fibers might be a valid biomarker for PD. However, several limitations will have to be addressed to allow its broad clinical application. First, consensus on the most accurate method of immunohistochemical staining and evaluation is needed in order to provide uniform data and determine clinical standards. Heterogeneous methods have been described including (1) the *α*-synuclein ratio: normalization of the total *α*-synuclein load to loss of intraepidermal nerve fibers, controlling for neural damage due to other causes, (2) employment of an antibody selective for the assumed pathological form of *α*-synuclein (phosphorylated *α*-synuclein), and (3) quantification of native *α*-synuclein without specificity for the pathological form or normalization to fiber loss [2, 7, 14]. While normalization to loss of intraepidermal nerve fibers and use of phosphorylated *α*-synuclein specific antibodies seem superior over native *α*-synuclein quantification, the optimal evaluation method remains to be determined. Second, *α*-synuclein aggregates were reported in distinct subtypes of skin autonomic fibers, comprising pilomotor and sudomotor fibers. However, which fiber type represents the most accurate diagnostic target needs to be answered and potential pathology in vasomotor fibers needs to be unveiled. Although two investigations assessing *α*-synuclein damage normalized to intraepidermal nerve fiber density reported most pronounced damage in pilomotor nerve fibers, potentially indicating high diagnostic value of this type of autonomic small fibers, independent replication of these observations is still warranted [2, 7]. Third, longitudinal multicenter studies in PD and other synucleinopathies that relate cumulative nerve fiber damage over time to progression of clinical symptoms are still lacking. Such studies are also necessary to confirm external validity of the applied techniques. Fourth, the influence of dopaminergic and neuroprotective treatment on the progression of autonomic synucleinopathic neuropathy needs to be elucidated [27]. Lastly, while immunohistochemical quantification of *α*-synuclein burden in small fibers reflects structural pathology, its implication on functional nerve fiber integrity is unknown.

## 8. Conclusion

Accumulating evidence supports an important pathogenic role of deposition of *α*-synuclein in cutaneous small nerve fibers in PD which seems to contribute even to development of the disease in its premotor stages. Quantitative analysis of this pathology in skin biopsies might facilitate early diagnostic discrimination among synucleinopathies, disease monitoring, and evaluation of response to neuroprotective treatment. Further research is needed to standardize evaluation techniques, characterize progression of pathology, and unveil the relation between functional and structural damage to small nerve fibers caused by *α*-synuclein accumulation, which might eventually answer the question whether we should perform skin biopsies routinely in patients suspected of having PD.
